# Is Smart Working Beneficial for Workers’ Wellbeing? A Longitudinal Investigation of Smart Working, Workload, and Hair Cortisol/Dehydroepiandrosterone Sulfate during the COVID-19 Pandemic

**DOI:** 10.3390/ijerph20136220

**Published:** 2023-06-24

**Authors:** Alessandra Falco, Damiano Girardi, Achim Elfering, Tanja Peric, Isabella Pividori, Laura Dal Corso

**Affiliations:** 1 FISPPA Section of Applied Psychology, University of Padua, 35131 Padua, Italy; 2 Institute of Psychology, University of Bern, 3012 Bern, Switzerland; 3 Department of Agricultural, Environmental and Animal Sciences, University of Udine, 33100 Udine, Italy

**Keywords:** smart working, COVID-19, workload, hair cortisol, dehydroepiandrosterone sulfate, biomarker, work-related stress, organizational wellbeing

## Abstract

Building on the job demands–resources (JD-R) and allostatic load (AL) models, in the present study we examined the role of smart working (SW) in the longitudinal association between workload/job autonomy (JA) and a possible biomarker of work-related stress (WRS) in the hair—namely, the cortisol–dehydroepiandrosterone sulfate (DHEA(S)) ratio—during the COVID-19 pandemic. Overall, 124 workers completed a self-report questionnaire (i.e., psychological data) at Time 1 (T1) and provided a strand of hair (i.e., biological data) three months later (Time 2, T2). Results from moderated multiple regression analysis showed that SW at T1 was negatively associated with the hair cortisol/DHEA(S) ratio at T2. Additionally, the interaction between workload and SW was significant, with workload at T1 being positively associated with the hair cortisol/DHEA(S) ratio at T2 among smart workers. Overall, this study indicates that SW is a double-edged sword, with both positive and negative consequences on employee wellbeing. Furthermore, our findings suggest that the hair cortisol/DHEA(S) ratio is a promising biomarker of WRS. Practical implications that organizations and practitioners can adopt to prevent WRS and promote organizational wellbeing are discussed.

## 1. Introduction

In recent years, technological advances and globalization have deeply affected the nature of work, fostering long working hours and making it more difficult to psychologically and physically detach from one’s professional obligations [1]. Specifically, the emergence of technologies such as emails, smartphones, and virtual meetings has facilitated the diffusion of alternative work arrangements, which provide workers with more flexibility in executing job tasks—in terms of time, space, and procedures—while, at the same time, contributing to blurred boundaries between work and private life [2]. Although the adoption of flexible work arrangements was ever-increasing in the pre-pandemic era [2], the COVID-19 crisis has caused a keen acceleration in this trend, as flexible work arrangements were widely adopted to limit the spread of SARS-CoV-2 (e.g., by reducing close social contacts with colleagues or customers) [3].

In the literature, different forms of alternative work arrangements are described [4,5,6], each with its own peculiarities—including, for example, telecommuting, remote work, telework, and smart working (SW). In their extensive review of the literature, Allen and colleagues defined telecommuting as “working some portion of time away from the conventional workplace, often from home, and communicating by way of computer-based technology” (p. 43) [4]. The authors also noted that remote work usually identifies more broadly “any form of work not conducted in the central office, including work at branch locations and differing business units” (p. 44) [4]. Additionally, according to the International Labour Organization, telework “refers to employees who use information and communications technologies to perform their work remotely”, usually from home—or from another location of their choice—on a regular or permanent basis (p. 8) [6].

In Italy, SW denotes a form of flexible work arrangement that implies an organization by phases, cycles, and objectives, the possible use of technological tools for carrying out the work activity, and the absence of constraints in time or place, thereby enabling remote working (Law 81/2017). Interestingly, SW was originally proposed to enhance competitiveness and foster work–life balance (Law 81/2017; see also [7]) However, to date, research has shown conflicting results, with SW being associated with both positive and negative consequences for workers’ wellbeing, before and during the COVID-19 pandemic [8,9,10,11,12]. 

With respect to its diffusion, SW was less common in Italy than in other European countries before the COVID-19 crisis [13], but during the pandemic the number of SW employees understandably increased, from 570,000 in 2019 to 6,580,000 in 2020 (+1054%) [14]. With the progress of the vaccination campaign and the gradual easing of some protective measures, the number of smart workers became 4.07 million in 2021 and 3.6 million in 2022. Interestingly, a slight increase to 3.63 million is expected in 2023 [15], which suggests that SW is part of the “new normal” [3].

Considering the growing spread of the phenomenon, and given its relevant consequences at a psychophysical (i.e., motivation, health, and wellbeing), social, and economic level [7,16,17], this study aimed at investigating the longitudinal association between SW and work-related stress (WRS), in terms of both perceived aspects of the job and the strain response, during the COVID-19 pandemic. Specifically, building on the job demands–resources (JD-R) model [18,19], we first hypothesized that workload and job autonomy (JA)—as perceived aspects of the job, in terms of job demands/resources, respectively—predict the strain response in the individual over time (i.e., three months later). Next, we focused on the role of SW in the aforementioned relationships. Considering its complex nature, we conceptualize SW as a broader “context” of work conditions, including at least one characteristic of SW that shapes remote employees’ work experience [3,9]. Accordingly, we hypothesized SW to predict the strain response in the individual over time. We also hypothesized that SW affects the longitudinal association between workload/JA and the strain response, as postulated by the JD-R model. Specifically, since smart workers may encounter difficulties in managing their daily workload and invest more effort in their work, we expected SW to exacerbate the positive association between workload and the strain response over time. Similarly, considering that smart workers may benefit more from JA because they may be better able to use discretion to organize their work effectively, we also expected that SW would enhance the negative association between JA and the strain response over time.

Additionally, to better understand the psychophysiological mechanisms underlying the relationship between job demands/resources, the strain response, and more serious long-term consequences of WRS (e.g., depression, cardiovascular disease) [20], as well as to reduce common method bias (CMB) [21], in this study we combined psychological and biological measures to determine perceived aspects of the job and the strain response, respectively. In doing so, we drew on the allostatic load (AL) model, which offers an integrative framework to understand the long-term impact of chronic/prolonged psychosocial stress on workers’ physical and mental health through cumulative physiological dysregulation [22,23]. The allostatic load, in fact, indicates the cumulative effects of everyday life events involving both ordinary and extraordinary challenges [24]. The possibility of knowing the allostatic load is beneficial in the assessment of the individual condition. A possible approach for determining allostatic load is the use of biological biomarkers [25]. Seeman et al. [26] identified different biological parameters such as cortisol and dehydroepiandrosterone sulfate (DHEA(S)), epinephrine, norepinephrine, cholesterol, glycosylated hemoglobin, resting systolic and diastolic blood pressure, body mass index, and waist–hip ratio reflecting the hypothalamic–pituitary–adrenal (HPA) axis activity [24,27]. Specifically, in this study, we focused on the cortisol-to-DHEA(S) ratio in hair, which can indicate allostatic load/chronic stress during the observation period between baseline and follow-up (see Section 2 for a detailed discussion). 

In summary, in this study, we longitudinally investigated the role of SW in the association between workload/JA and cortisol/DHEA(S) ratio, as a possible biomarker of WRS. By doing so, we contribute to the literature in at least two ways: First, by conceptualizing SW as a “context” that shapes the remote workers’ work experience [3], we aimed to investigate whether and how two well-known job demands/resources among in-person workers (i.e., workload and JA) can contribute to smart workers’ health and wellbeing. In addition to possible theoretical advancements, this may also have practical implications for organizations and managers, who can foster smart workers’ wellbeing and productivity by designing remote work appropriately to match their specific needs and expectations. Next, previous studies have largely shown that SW may have both beneficial and adverse consequences for worker health and wellbeing [28,29,30,31]. However, past research was largely based on self-reported, cross-sectional data [28,30] and did not consider physiological mechanisms associated with SW over time [32] (with some exceptions) [33]. By using a multimethod longitudinal research design, we aimed to make a sound contribution to the field. In the following sections of this article, we provide a detailed description of the theoretical framework behind our hypotheses. First, we outline the theoretical background of the study, briefly describing the JD-R and AL models. Next, we concentrate on the study hypotheses and their theoretical underpinnings, with a focus on the role of SW. Finally, in Section 2, we describe in detail the cortisol/DHEA(S) ratio in hair as a possible biomarker of WRS.

### 1.1. Job Demands/Resources and the Allostatic Load Model

The JD-R model [18,19,34] is a flexible theoretical model that synthesizes knowledge from previous theories of WRS and motivation, providing a thorough understanding of employee wellbeing and performance [19]. A core assumption of the JD-R model is that job characteristics can be classified either as job demands or job resources. The former are those “physical, psychological, social, or organizational aspects of the job that require sustained physical and/or psychological effort and are therefore associated with certain physiological and/or psychological costs “ (p. 274) [34] and include, among others, workload and interpersonal conflict [19]. Job resources—including, for example, job autonomy and social support [19]—are “those physical, psychological, social, or organizational aspects of the job that are functional in achieving work goals, reduce job demands and the associated physiological and psychological costs, or stimulate personal growth, learning, and development” (p. 274) [34]. According to the health impairment process of the JD-R model, the frequency and/or severity of job demands, by requiring effort, depletes workers’ physical, emotional, and cognitive resources, possibly leading to exhaustion and psychophysical strain (i.e., psychological and physical symptoms related to WRS) over time [35,36,37]. Similarly, a lack of job resources thwarts the achievement of one’s goals at work, which may lead to exhaustion/psychophysical strain over time [36]. 

However, as previously noted by Schaufeli and Taris [36], the JD-R model is a general, descriptive model that defines the relationships between categories of constructs, such as job demands/resources and health/motivation, without focusing on the underlying psychological or physiological mechanisms. Interestingly, physiological costs are mentioned in the definition of both job demands and resources, but research on the physiological processes involved in the long-term association between chronic/prolonged stressful situations at work, psychophysical strain, and more serious long-term consequences of WRS (e.g., depression, cardiovascular disease) [20] is still limited [34,38]. Hence, we also build on the AL model [22,39]—which has previously been applied to WRS [20] and integrated with the JD-R model [40,41]—to provide theoretical support for the associations hypothesized in this study. According to the AL model, the exposure to stressful situations (at work) triggers the secretion of so-called primary mediators, which include stress hormones (e.g., cortisol) [42,43] and pro-/anti-inflammatory cytokines. When a worker faces chronic or repeated stressful conditions, and when recovery is incomplete [44], primary mediators are activated chronically or repeatedly. Over time, this may lead to secondary mediators (e.g., increased blood pressure) and, eventually, allostatic overload and psychological or physical diseases—including, for example, depression or cardiovascular disease [20]. In summary, the AL model offers an integrative framework to understand the impact of chronic/prolonged psychosocial stress—including WRS—on workers’ physical and mental health through cumulative physiological dysregulation [22,23].

### 1.2. The Present Study

In the literature on WRS and organizational wellbeing, workload and JA are identified as central job demands and resources in the general working population [35,45,46,47]. Furthermore, workload and JA play a pivotal role in several theoretical models, such as the Demand–Control–Support model [48,49], the Effort–Reward Imbalance model [50], the Health and Safety Executive’s Management Standards [51], the Job Characteristics Model [52], and the Vitamin Model [53], as well as the JD-R model [18]. Generally, two related facets of workload are identified in the literature, namely, quantitative and qualitative workload [54,55]. While the former pertains to the quantity of work to be carried out in a given period of time, the latter refers to the “difficulty or complexity of the job, for which the worker is not trained or does not have enough resources to deal with” (p. 2) [56]. Consistent with the health impairment process of the JD-R model, workload—both quantitative and qualitative—requires effort to meet one’s job requirements and drains individuals’ physical and mental resources, such as energy, concentration, or time [45]. Being constantly exposed to high workloads, coupled with inadequate recovery opportunities [44], leads to the progressive depletion of resources without adequate replenishment, and this may result in psychophysical strain over time [44,57]. Both cross-sectional and longitudinal research provides evidence of a relationship between workload and adverse individual outcomes of WRS, including psychophysical strain and exhaustion [35,45,46]. Overall, in line with the health impairment process of the JD-R, and consistent with prior empirical studies, we hypothesized that the workload at Time 1 (T1) would be positively associated with the hair cortisol/DHEA(S) ratio at Time 2 (T2), three months later.

**Hypothesis** **1** **(H1).** *Workload at T1 will be positively associated with hair cortisol/DHEA(S) ratio at T2.*

Job autonomy has been conceptualized as the “extent to which a job allows freedom, independence, and discretion to schedule work, make decisions, and choose the methods used to perform tasks” (p. 1323) [58]. As a job resource, JA is functional in the effective performance of work tasks and the achievement of one’s goals at work, thereby contributing to prevent psychophysical strain and exhaustion over time [36]. Similarly, JA plays a central role in employee health and wellbeing, because greater autonomy corresponds to more opportunities to cope effectively with stressful situations at work [59,60]. For example, when workers are allowed to control their work, they may be flexible in adapting to unexpected situations, or they may develop new strategies to overcome temporary challenges or difficulties and complete their tasks as required [61,62]. Furthermore, JA may help workers to improve other valuable resources at work, such as self-efficacy and perceived control [63], which can protect them from negative consequences of WRS [64,65,66]. In line with this reasoning, past cross-sectional and longitudinal research has shown JA to be negatively associated with psychophysical strain and exhaustion [67,68,69]. Hence, we hypothesized that JA at T1 would be negatively associated with the hair cortisol/DHEA(S) ratio at T2.

**Hypothesis** **2** **(H2).** *Job autonomy at T1 will be negatively associated with hair cortisol/DHEA(S) ratio at T2.*

The next three hypotheses concern the role of SW in the longitudinal relationship between workload/JA and hair cortisol/DHEA(S) ratio. Smart working implies a radical change in physical and psychosocial working conditions, with potentially relevant effects on individuals’ wellbeing, productivity, and overall quality of working and private life [28,29]. Although previous research has shown SW to have both favorable and adverse consequences [28,29,30,31,70], recent reviews suggest an overall beneficial impact [29], particularly for some professionals, e.g., knowledge workers (i.e., “employees who have to acquire, create and apply knowledge for the purposes of their work”, p. 51) [70]). Similarly, empirical research suggests positive outcomes of SW among specific workers’ profiles: for example, remote working improved depressive symptoms among working women with young children [71], and the extent of remote working was positively associated with job performance among those who hold complex jobs or jobs involving low levels of interdependence [72]. A possible explanation is that SW helps workers to maintain or replenish resources such as time, physical/psychological energy, or capacities, thereby contributing to prevent resource depletion, exhaustion, and negative health consequences over time [73,74]. For example, SW may avoid or reduce office interruptions, thereby preserving the level of concentration necessary to complete one’s tasks [75]. Other potentially beneficial aspects of SW include reduced commuting time, especially for those who frequently work remotely (e.g., more than three days per week) [76]; increased opportunities for leisure, particularly among teleworkers without children [77]; the possibility to work according to one’s own biorhythms [31]; and the adoption of health behaviors [32], including an improved sleep pattern [78], increased physical activity [79], and healthier eating [80]. Overall, in the light of previous theoretical reasoning and empirical evidence, we tentatively hypothesized that SW would be negatively associated with hair cortisol/DHEA(S) ratio over time.

**Hypothesis** **3** **(H3).** *Smart working at T1 will be negatively associated with hair cortisol/DHEA(S) ratio at T2.*

Next, in this study, we hypothesized that SW might affect the longitudinal association between job demands/resources (in terms of workload/JA) and hair cortisol/DHEA(S) ratio. First, smart workers may find it difficult to manage their day-to-day workload due to technology issues (e.g., failures of network connections) [81] or lack of adequate technological equipment [82], the communication they receive via email (high quantity/poor quality) [83], or an excessive quantity of virtual meetings, which might give rise to Zoom fatigue [84,85,86]. This may eventually lead to information overload [87] and reduced control over one’s own workflow [88], feelings of being overwhelmed by technology (a dimension of technostress) [89,90], and impaired recovery experiences [91]. Additionally, smart workers may encounter difficulties in concentrating on their work tasks because of demands or frequent interruptions from the family domain [8]. As a result, they usually have to carry out several tasks at the same time—both work- and family-related—or switch between different tasks (i.e., multitasking) [92]. Overall, this implies that smart workers may exert greater effort to perform their work tasks, with higher psychophysiological cost to the individual and, ultimately, higher levels of exhaustion and psychophysical strain over time (i.e., the health impairment process of the JD-R model). Hence, we hypothesized that SW would moderate the positive association between workload at T1 and hair cortisol/DHEA(S) ratio at T2, which is expected to be stronger for smart workers.

**Hypothesis** **4** **(H4).** *Smart working at T1 will moderate the positive association between workload at T1 and hair cortisol/DHEA(S) ratio at T2, which is expected to be stronger for smart workers.*

The last hypothesis concerns SW and JA. By being less bounded by office routine and direct supervision, and also given the extensive adoption of new technologies, smart workers may benefit more from JA because they may be better able to use discretion to organize their work effectively [93]. For example, smart workers may decide to postpone a task to a more preferable time of the day (e.g., in the evening), or they may modify their task schedule or method of execution (e.g., modifying daily tasks to make better use of new technologies or modifying technologies to perform a task more efficiently) [94], thereby mitigating fatigue and exhaustion while fostering task performance [93,95]. Similarly, smart workers can take advantage of their autonomy to better manage demands from the family domain or to engage in household or leisure activities, leading to an improved work–life balance and reducing their need for recovery [5,96]. Accordingly, our last hypothesis was that the negative association between JA at T1 and hair cortisol/DHEA(S) ratio at T2 would be stronger for smart workers—that is, JA contributes to preventing negative consequences of WRS (e.g., psychophysical strain), especially among smart workers.

**Hypothesis** **5** **(H5).** *Smart working at T1 will moderate the negative association between JA at T1 and hair cortisol/DHEA(S) ratio at T2, which is expected to be stronger for smart workers.*

## 2. Materials and Methods

### 2.1. Participants and Procedures

The study was carried out in Italy during the COVID-19 pandemic and involved a sample of workers from different organizations. Participants were recruited using a snowball procedure and were invited to take part in a longitudinal study about their work experience and biomarkers of WRS in the hair. Workers were motivated to participate by explaining the general aims of the research and its relevance. They were also informed that participation was voluntary and confidential, and that they could withdraw at any time. Two waves of data collection were conducted. The first wave (i.e., T1) started in mid-March 2022, whereas the second wave (i.e., T2) took place in mid-June 2022, with a three-month interval between measurement occasions. Briefly, participants were invited to complete an online questionnaire at T1, which was aimed at determining the psychological constructs under investigation. Participants were also informed that they would be required to collect a biological sample—namely, a strand of hair approximately 3 cm long—three months later (i.e., T2). 

Upon acceptance, participants were given a link to the informed consent form and the questionnaire, so that all participants provided written informed consent before filling out the self-report instrument. Additionally, participants were also provided with an alphanumeric identification code, which was necessary to match psychological and biological data collected over time. At T2, participants were provided with detailed instructions about the collection of biological samples (including a brief video tutorial). Furthermore, at both T1 and T2, the participants were invited to complete a short online questionnaire aimed at collecting information useful for the subsequent analysis of the hair samples (e.g., pregnancy status, medication intake). Next, at T2, a hair strand measuring at least 3 cm was collected noninvasively from the vertex posterior of the head, cut as close to the scalp as possible. Hormone concentrations—in pg/mg—were determined from the first scalp-near 3 cm hair segment, reflecting the cumulative concentrations of cortisol and DHEA(S) over the three-month period between the two measurement occasions. The project was approved by the Ethical Committee for Psychological Research of the University of Padua. 

Overall, 150 workers were invited to take part in the study; 137 participants completed the questionnaire at T1, and 124 (90.5%) also provided hair samples at T2. No differences emerged in the main demographics or study variables between study participants and dropouts (*n* = 13). The sample included 91 women (73.4%) and 33 men (26.6%), with a mean age of 39.9 years (SD = 13.6). With respect to work experience, 51 workers (41.1%) had less than 5 years of working seniority, whereas 48 (38.7%) had more than 10 years of working seniority. Seventy-eight workers (62.9%) held a permanent contract, while forty-six (37.1%) held a temporary contract. Concerning marital and parental status, 72 workers (58.1%) were married or cohabitating, and 50 (40.3%) had children. Finally, concerning SW, there were 84 in-person workers (67.7%) and 40 smart workers (32.3%).

### 2.2. Psychological Measures

With respect to psychological data at T1, the questionnaire included the following self-report measures:

Workload was assessed using seven items reflecting both qualitative and quantitative workload [35,54]. The scale was taken from the Q_u_–Bo Test—a self-report, standardized instrument developed for the Italian context [97]. The scale items were formulated to capture the general level of workload and used a 6-point response scale ranging from 1 (strongly disagree) to 6 (strongly agree), where higher scores reflect greater workload. A sample item is “Your job requires you to work constantly under pressure”. Cronbach’s alpha was 0.85 in this study.

Job autonomy was determined using a scale taken from the Q_u_–Bo Test [97]. The scale included three items that captured the general level of JA and used a 6-point response scale ranging from 1 (strongly disagree) to 6 (strongly agree), where higher scores indicate greater JA. A sample item is “Your job allows you to autonomously decide the pace of work”. Cronbach’s alpha was 0.90 in this study.

Smart working was assessed by asking participants to indicate whether they worked in person or remotely, in whole or in part (i.e., smart worker).

### 2.3. Biological Measures

In this study, we considered the hair cortisol/DHEA(S) ratio as a possible biomarker of WRS. Specifically, cortisol—a biomarker of HPA axis activity—helps the body in adapting flexibly to environmental challenges [98]. Likewise, DHEA(S) is a neuroactive steroid, potent modulator of neurogenesis, neuronal growth, and differentiation, and neuroprotector that counteracts the effects of glucocorticoids [27,99]. Therefore, using cortisol and DHEA(S) individually is certainly useful for understanding an individual’s state, but the cortisol/DHEA(S) ratio—an index of the catabolic/anabolic balance—may be more informative and helpful than their absolute concentrations [99]. Cortisol and DHEA(S) have antagonistic effects on one another, allowing us to monitor the effects of psychological processes on the long-term HPA axis activity. Interestingly, it has been proposed that the ratio between cortisol and DHEA(S) reflects an imbalance in the HPA axis associated with chronic stress [100,101]. An altered ratio has been associated with psychological outcomes such as higher anxiety, mood disturbance, confusion, and poorer cognitive performance [27,102]. Mental health may also be negatively affected by a high cortisol/DHEA-S ratio. There is also convincing evidence that this ratio may serve as a robust indicator of immune function. A high cortisol/DHEA(S) ratio has been observed in humans suffering from severe injuries and illnesses and can also be used to predict the risk of infection or death [102]. This ratio has also been considered as a possible biomarker of adverse psychosocial stress, including WRS [103,104]. 

The cortisol and DHEA(S) concentrations can be measured by a variety of matrices, each reflecting a specific timeframe of HPA axis activation. Measurements of these hormones in blood serum, saliva, and urine provide snapshot information of exposure, while retrospective determination can be achieved through a single strand of hair [105]. A strength of hair measurement is that, in line with the AL model, it allows the retrospective assessment of cumulative hormone concentrations over several months (e.g., three months, as in the current study), which reflect an individual’s physiological activation in response to the exposure to chronic/prolonged stressful events over the same time period [106,107]. Hair sampling is not invasive or painful, samples can be stored at room temperature for extended periods of time [108], and there is a low susceptibility to confounding factors (e.g., circadian and ultradian rhythmicity) [108].

#### 2.3.1. Hair Collection 

In regard to biological data at T2, hair was collected from the vertex posterior region of the head, since it has been found that this area has the greatest growth synchrony [109]. The collected hair strand represents the hair growth in the period between T1 and T2 based on an average hair growth of 1 cm/month [109]. Each sample was stored in a paper envelope at room temperature and protected from UV rays until processing.

#### 2.3.2. Sample Preparation 

Twenty-five milligrams of hair was weighed, and each hair strand was washed twice using H_2_O for 3 min and then, in agreement with Davenport et al. [110] twice with isopropanol for 3 min. These stages allowed us to minimize the risk of extracting cortisol from outside the hair and ensure the removal of sweat and sebaceous secretions from the external surface of the hair. Steroids were extracted by incubating each specimen for 16 hours in methanol at 37 °C. Next, the liquid in the vial was evaporated to dryness at 37 °C under an airstream suction hood. The dried residue was then resuspended in 1.2 mL of ELISA buffer (50 mM phosphate buffer, pH 7.4, 0.4% BSA, 0.5 M NaCl).

#### 2.3.3. Hair Hormone (Cortisol and DHEA(S)) Analysis

The concentrations of cortisol and DHEA(S) were measured using an in-house enzyme-linked immunosorbent assay (ELISA), as described already for another hormone (progesterone) by Comin et al. [111]. In brief, microplates were coated with anti-rabbit-IgG antibody. After an overnight incubation, the plates were washed 5 times with washing buffer. Aliquots of cortisol or DHEA-S standards, quality-control extract, and hair test extracts were added to the microplate wells. The cross-reactivities of the anti-cortisol antibody with other steroids were as follows: cortisol 100%, cortisone 4.3%, corticosterone 2.8%, 11-deoxycorticosterone 0.7%, 17-hydroxyprogesterone 0.6%, dexamethasone 0.1%, progesterone, 17-hydroxypregnenolone, DHEAS, androsterone sulfate and pregnenolone < 0.01%. The cross-reactivities of the DHEA(S) antibody with other steroids were as follows: DHEA-S 100%, 5-androsten-3-ol-17-one (dehydroepiandrosterone, DHEA) 76.6%. Anti-cortisol or anti-DHEA(S) antibody diluted 1:32,000 and 1:80,000, respectively, in ELISA buffer was added along with the cortisol- or DHEA-S-peroxidase conjugate diluted 1:6000 or 1:10,000, respectively, in ELISA buffer. Plates were incubated overnight and then washed 5 times in washing buffer to remove any unbound cortisol or DHEA(S). The amount of bound conjugate was quantified by adding the chromogenic substrate. The plates were incubated for 30 min in darkness at room temperature (23 °C). The reaction was stopped with 2 M H_2_SO_4_. Absorbance was read at 450 nm using a plate reader (EnSight Multimode Plate Reader, Perkin-Elmer Life Science, Boston, MA, USA). The intra- and inter-assay coefficients of variation were 6.3% and 12.2%, and 9.9% and 15.2%, for cortisol and DHEA(S), respectively. The sensitivities of the assays were 9.4 and 5.4 pg/mL for cortisol and DHEA(S), respectively. The relationships between hair cortisol and hair DHEA(S) and their respective standard curves (parallelism), as determined through linear regressions, were linear, with correlation coefficients of r = 0.99. The models were described by the equations y = 0.99x − 0.15, and y = 1.03x − 2.71 for cortisol and DHEA(S), respectively. The recovery rate was 99.8 ± 14.5% and 110.9 ± 4.1% (mean ± SD) for cortisol and DHEA(S), respectively.

### 2.4. Data Analysis

First, the psychometric properties of the psychological measures (i.e., self-report questionnaires) were investigated using a confirmatory factor analysis (CFA), which was carried out using maximum likelihood with robust standard errors and a scaled test statistic as an estimator [112]. In a two-factor model, workload and JA at T1 were measured by the respective scale items. To assess the model fit, we considered the scaled chi-squared test along with the following fit indices: the root-mean-square error of approximation, the comparative fit index, and the standardized root-mean-square residual (hereafter RMSEA, CFI, and SRMR, respectively). A nonsignificant χ^2^, as well as values below 0.08 for RMSEA and SRMR, and values above 0.90 for CFI, indicated an acceptable fit [113]. The reliability of each scale was assessed using composite reliability, whose values greater than 0.70 suggest satisfactory reliability [114].

Next, moderated multiple regression analysis was used to test the relationships hypothesized in the study [115]. In Model 1 (M1), the hair cortisol/DHEA(S) ratio at T2 was regressed on gender, age, workload, JA, and SW at T1. In Model 2 (M2), two interaction terms were also included: the first between workload and SW, and the second between JA and SW. If significant interactions emerged, then a simple slope analysis was carried out to investigate whether the relationships between predictors at T1 (i.e., workload or JA) and cortisol/DHEA(S) ratio at T2 were significant across in-person vs. SW. Furthermore, significant interactions were plotted as described by Aiken and West [115]. Finally, as an ancillary analysis, we estimated an additional Model 3 (M3), in which we also included the interaction between workload and JA [116]. The independent variables included in M1/M2/M3 (excluding dichotomous variables) were mean-centered, to enable easier interpretations of the results. Additionally, a log-transformation of the cortisol/DHEA(S) ratio was used, which was computed as follows: log_10_(Cortisol/DHEA(S)) = log_10_(Cortisol) − log_10_(DHEA(S)). The ratio was log-transformed to improve its distribution and symmetry, thereby ensuring the appropriateness of the subsequent parametric tests (e.g., regression analysis) [117]. All regression models were estimated controlling for the effects of gender and age [118], as previous research has shown an association between hair cortisol/DHEA(S) concentrations and these demographic variables [104,119,120,121]. The results described above are shown in the manuscript. Additionally, as previous research has suggested an association between hair cortisol/DHEA(S) concentrations and pregnancy status [122], as well as medication intake [123], we further investigated the role of these variables, and the main models discussed in the manuscript (M1 and M2) were estimated controlling for the effects of pregnancy status and medication intake, where necessary. These results are available in the Supplementary Materials. Statistical analyses were conducted using R version 4.2.1 [124], as well as the lavaan R package version 0.6–13 for CFAs [112].

## 3. Results

### 3.1. Confirmatory Factor Analysis

First, the CFA model showed a moderate fit to the data: χ^2^(34) = 67.08, *p* < 0.001; RMSEA = 0.089, CFI = 0.923, SRMR = 0.086. An inspection of the modification indices suggested that the error covariance between items 2 and 3 of workload should be freely estimated. This is conceivable, given that these items both reflect quantitative workload (i.e., the amount of work to be done in a given time) [35,54] and share similar wording. A second CFA was performed, and the fit indices showed an acceptable fit to the data: χ^2^(33) = 47.98, *p* = 0.05; RMSEA = 0.060, CFI = 0.965, SRMR = 0.076. The revised model also showed a better fit to the data: ∆χ^2^(1) = 17.43, *p* < 0.001. Finally, the composite reliability was 0.84 for workload and 0.91 for JA. All in all, the self-report questionnaires administered at T1 showed adequate psychometric properties.

### 3.2. Preliminary Analysis

Descriptive statistics were first examined for the study variables. The mean biomarker concentration in hair was 27.85 pg/mg (*SD* = 42.79 pg/mg) for cortisol and 65.32 pg/mg (*SD* = 50.7 pg/mg) for DHEA(S). Log cortisol/DHEA(S) ratio (*M* = −0.45, *SD* = 0.38), workload (*M* = 3.88, *SD* = 1.11), and JA (*M* = 3.80, *SD* = 1.58) showed values of univariate skewness and kurtosis that fell within the range of ±2.0 and ±7.0, respectively [125]. Correlation analysis showed a positive albeit marginally significant association between workload and JA (*r* = 0.17, *p* = 0.06), whereas zero-order correlations between log cortisol/DHEA(S) ratio and workload (*r* = 0.07, *p* = 0.46) or JA (*r* = 0.10, *p* = 0.28) were not significant. Interestingly, there was a significant difference in log cortisol/DHEA(S) ratio by SW, with higher levels among in-person workers (*M* = −0.39, *SD* = 0.37) compared to smart workers (*M* = −0.58, *SD* = 0.37) (*t*(75.83) = 2.58, *p* = 0.01, Cohen’s *d* = 0.50) [126]. Conversely, there was no significant difference in workload between in-person workers (*M* = 3.79, *SD* = 1.17) and smart workers (*M* = 4.05, *SD* = 0.98) (*t*(90.42) = −1.28, *p* = 0.20, Cohen’s *d* = 0.24). Similarly, there was no significant difference in JA between in-person workers (*M* = 3.71, *SD* = 1.69) and smart workers (*M* = 3.98, *SD* = 1.31) (*t*(96.92) = −0.95, *p* = 0.34, Cohen’s *d* = 0.17). Next, with respect to control variables, there was a marginally significant difference in log cortisol/DHEA(S) ratio across gender, with relatively higher levels in females (*M* = −0.41, *SD* = 0.32) compared to males (*M* = −0.57, *SD* = 0.48) (*t*(42.98) = 1.83, *p* = 0.07, Cohen’s *d* = 0.39). Finally, there was a positive association between log cortisol/DHEA(S) ratio and age (*r* = 0.33, *p* < 0.001).

### 3.3. Hypothesis Testing

The results of the regression analyses are presented in Table 1. In both M1 and M2, a positive vs. negative association between predictors at T1 and hair cortisol/DHEA(S) ratio at T2 would be interpreted as a health-threatening vs. protective effect, based on the conceptualization of the ratio as a biomarker of WRS. In M1, the predictors at T1 accounted for 18.6% of the variance in the log cortisol/DHEA(S) ratio at T2 (*R*^2^ = 0.19, *F*(5, 118) = 5.39, *p* < 0.001). In this model, gender (*b* = −0.13, *SE* = 0.07, *p* = 0.07) and age (*b* = 0.01, *SE* = 0.00, *p* < 0.01) at T1 were associated with log cortisol/DHEA(S) ratio at T2, although the association was marginally significant in the former case. Workload (*b* = 0.03, *SE* = 0.03, *p* = 0.27) and JA (*b* = 0.01, *SE* = 0.02, *p* = 0.66) at T1 were not associated with log cortisol/DHEA(S) ratio at T2. Hence, H1 and H2 were not supported. Smart working at T1 (0 = in-person working, 1 = SW) was negatively associated with log cortisol/DHEA(S) ratio at T2 (*b* = −0.18, *SE* = 0.07, *p* < 0.01), and H3 was supported. 

**Figure 1:**
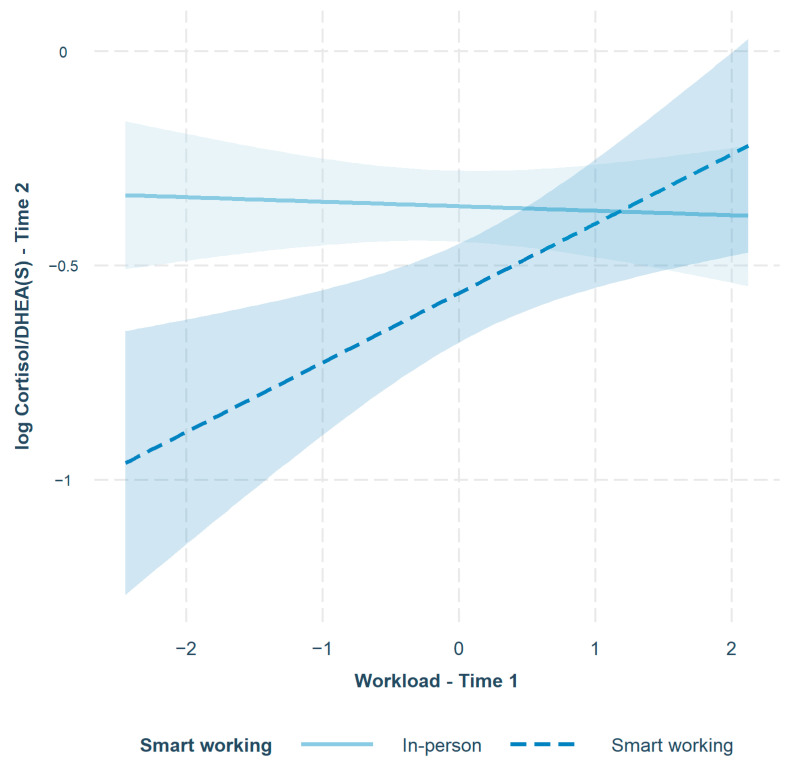
The moderating role of smart working in the relationship between workload and log cortisol/DHEA(S) ratio over time. The 95% confidence intervals around each slope are shown in blue.

In M2, the interaction terms accounted for an additional 5.2% of the variance in log cortisol/DHEA(S) ratio at T2: *F*_change_(2, 116) = 3.96, *p* = 0.02, *f*^2^ = 0.07 [126]. The interaction between workload and SW was significant (*b* = 0.17, *SE* = 0.07, *p* < 0.01), whereas the interaction between JA and SW was not (*b* = 0.03, *SE* = 0.05, *p* = 0.52). Simple slope analysis showed that the association between workload at T1 and log cortisol/DHEA(S) ratio at T2 was positive and significant for smart workers (*b* = 0.16, *SE* = 0.06, *p* < 0.01), but not significant for in-person workers (*b* = −0.01, *SE* = 0.03, *p* = 0.75). The interaction between workload and SW is shown in Figure 1. Smart working enhanced the positive association between workload at T1 and log cortisol/DHEA(S) ratio at T2. Overall, Hypothesis 4 was supported, but Hypothesis 5 was not. Finally, in M3, the interaction between workload and JA was not significant: *F*_change_(1, 115) = 0.13, *p* = 0.72. Summarizing, SW was associated with a lower (better) hair cortisol–DHEA(S) ratio, so SW is beneficial for workers’ wellbeing overall, but if work stressors (i.e., workload) increase the advantage of SW is lost.

## 4. Discussion

In this study, we longitudinally investigated the role of SW in the WRS process. Specifically, building on the JD-R and the AL models, we examined the associations over time between two relevant job demands/resources in the general working population—namely, workload/JA, and cortisol/DHEA(S) ratio in the hair—as a possible biomarker of WRS. We also investigated the role of SW in the aforementioned process and relationships. Based on theoretical reasoning and past empirical results, we first hypothesized SW to have an overall beneficial impact on workers’ health and wellbeing, being negatively associated with the hair cortisol/DHEA(S) ratio over time. Next, we suggested that SW may intensify the positive association between workload and hair cortisol/DHEA(S) ratio, since smart workers may encounter difficulties in completing their work tasks. Finally, we hypothesized that SW may also intensify the negative association between JA and hair cortisol/DHEA(S) ratio, so that JA contributes to preventing the negative consequences of WRS, especially among smart workers.

The results partially supported our predictions. In contrast to our expectations, neither workload nor JA at T1 was associated with hair cortisol/DHEA(S) at T2 (i.e., three months later). Interestingly, SW was negatively associated with hair cortisol/DHEA(S) ratio over time. Furthermore, the interaction between workload and SW was significant, with the association between workload at T1 and hair cortisol/DHEA(S) ratio at T2 being positive and significant for smart workers. Finally, the interaction between JA and SW was not significant.

### Theoretical Implications

We think that our research provides a valuable contribution to the existing literature on SW, with both theoretical and practical implications. To begin with, in this study, we conceptualized SW as a “context” that affects the meaning of work and the ability of workers to manage their professional obligations effectively, including with respect to the family and private life domains [3]. From this perspective, SW does not necessarily influence workers’ health, wellbeing, and productivity though a different perception of work characteristics (e.g., increased workload, reduced quality of relationships with supervisor/colleagues) [5]. Rather, to enhance wellbeing and job performance, it is important that work characteristics (i.e., job demands/resources) fit the specific, flexible work arrangement as well as remote workers’ specific needs and expectations. As noted by Wang and colleagues [3], this approach is especially useful in the context of the COVID-19 crisis, when SW was often a necessity rather than an option (depending on the measures adopted to contain the spread of the SARS-CoV-2 virus), and the meaning of specific job demands/resources is deeply affected by the exceptional COVID-19 crisis.

It is interesting to note that, in this study, smart workers did not show higher levels of workload or JA compared to in-person workers (see Section 3). Conversely, and consistent with the proposed theoretical perspective, SW emerged as a complex phenomenon, with in-depth, wide-ranging effects on employees’ health and wellbeing [32]. On the one hand, by being negatively associated with hair cortisol/DHEA(S) ratio, SW seemed to be related to lessened psychophysical strain over time. This result is consistent with recent empirical evidence, showing that SW may have both favorable and adverse consequences, but with an overall beneficial effect on employees’ wellbeing [28,29,30,70]. By reducing commuting time, favoring the adoption of healthy behaviors (e.g., improved sleep patterns), and increasing opportunities for leisure activities [32,76,77,78], SW may help workers to maintain or replenish resources (e.g., time, energy) and to prevent negative health consequences associated with resource exhaustion over time [73,74]. On the other hand, SW exacerbated the positive association between workload at T1 and hair cortisol/DHEA(S) ratio at T2, with this association being positive and statistically significant for smart workers. A possible explanation is that SW implies both an extension and an intensification of work [127]. Because smart workers may encounter difficulties in managing their workload effectively [81,83,84,128], they end up dedicating more time and effort—physical and mental—to their work, in order to meet job requirements and achieve their objectives. This may result in sustained psychophysiological activation and, ultimately, negative health outcomes [22,44]. Interestingly, these findings are consistent with a recent longitudinal study showing a positive association between workload and exhaustion—a central feature of job burnout—among smart workers over time [9]. All in all, our findings make a contribution to the literature by showing that SW can be seen as a double-edged sword for employee wellbeing [129]. By increasing the flexibility in defining the spatiotemporal boundaries of the work and the massive adoption of new technologies, SW generates opportunities for employees to harmoniously integrate work and non-work activities but, at the same time, it also contributes to hindering the effective achievement of one’s work goals through the disruption of employees’ workflow and information overload [130,131,132], with opposite effects on individual wellbeing.

Second, recent reviews on the outcomes of SW have sometimes shown conflicting results [28,29,30,31]. However, it should be noted that past empirical research was largely based on cross-sectional data [28,30] (with some exceptions, e.g., [133,134]), which precluded conclusions about the direction of the observed relationships between SW, job demands/resources, and mental/physical health [70]. Similarly, most previous studies solely included self-report measures and did not consider physiological measures (for notable exceptions, see [135,136]), so our understanding of the physiological processes involved in the aforementioned associations is still limited [34,137]. Additionally, cross-sectional, single-method research is also susceptible to CMB [21,138]. Therefore, to fill a gap in the literature, as well as to contain CMB, we used a multimethod longitudinal research design that combined psychological and biological measures. By showing an association between SW, workload, and cortisol/DHEA(S) ratio in the hair, our results provide useful insight into physiological mechanisms potentially involved in the association between stressful working conditions and stress-related health impairment over time [139,140]. Specifically, the exposure to high workloads, coupled with an impaired ability to manage one’s job demands among smart workers (e.g., due to information overload or disrupted workflow), may be associated with a sustained activation of the HPA axis [141,142]. Over time, this sustained activation of so-called primary mediators [22] may result in an imbalance of the HPA axis, as reflected by an elevated cortisol–DHEA(S) ratio in hair [100,101]. At the same time, the opportunities for smart workers to more harmoniously integrate the work and non-work domains may help them to inhibit the sustained activation of the HPA axis, which helps to counterbalance—at least partially—the effects associated with suboptimal workload management [44]. 

Overall, our results are rather consistent with prior research in the field, although with some differences. For example, studies on hair cortisol concentration (HCC) showed that working from home was associated with greater maternal HCC levels during the COVID-19 pandemic [136], whereas van der Meij and colleagues found that HCCs were higher in a high-workload sample compared to a normal-workload sample (i.e., workers following an executive management program outside their normal jobs vs. those with regular jobs) [142]. Research on cortisol/DHEA ratio has shown mixed results. For example, a study by Kim and colleagues showed that the molar cortisol/DHEA ratios on Sunday were significantly lower than those on workdays among full-time working individuals who underwent saliva sample collections for seven consecutive days [143]. Similarly, a recent work by Ledford and colleagues found that serum levels of DHEA/cortisol ratio—as indicative of physiological resilience—was associated with one’s ability to successfully complete the first phase of a military special operations training course [144]. Conversely, Ota and colleagues did not find an association between effort–reward imbalance or overcommitment to work and daytime salivary cortisol, DHEA, or cortisol/DHEA ratio among female nursery schoolteachers [145]. In light of the complex picture from previous research, two main peculiarities of the biological assessment carried out in this study need to be acknowledged: First, traditional assessment in saliva or serum mostly captures acute, short-term stress responses [120,146]. However, in line with the AL model, in this study we focused on hair concentrations of cortisol and DHEA(S) as a retrospective measure of the sustained, long-term HPA activity associated with chronic/prolonged stress [147,148]. Second, given the mixed findings concerning the association between WRS and hair cortisol [106,149,150,151] (see also [148] for a review), in this study we specifically focused on the ratio between hair concentrations of two stress-related hormones—namely, cortisol and DHEA(S)—as a promising biomarker [103,104] that reflects an imbalance in the HPA axis associated with chronic/prolonged stress [100,101]. 

Contrary to our expectations, by not being negatively associated with the cortisol/DHEA(S) ratio, JA did not seem to play a role in preventing negative outcomes of WRS. This unexpected result might be explained in the light of recent research suggesting that job resources are not necessarily and universally beneficial, but that the value of a resource may depend on its levels or the context in which a specific resource occurs [152]. With respect to the levels of JA, it should be noted that the study participants were mostly white-collar workers (77.4%) doing intellectual (e.g., freelancers, managers, office workers, or teachers) rather than manual work. Hence, it is possible that for these workers the beneficial effect of JA was somewhat limited, because autonomy is already an intrinsic component of their knowledge-based jobs, as reflected by the fairly high JA scores reported (*M* = 4.01, *SD* = 1.58 on a response scale ranging from 1 to 6) [153]. Additionally, according to the vitamin model [53], the beneficial effect of a job resource can rise to a certain level, and then a further increase in the resource may have no additional effect or, alternatively, a detrimental effect. From this perspective, the lack of association between JA and cortisol/DHEA(S) ratio also among smart workers is not surprising, given that those working remotely likely had occupations characterized by a good deal of autonomy even before they took advantage of SW [154], meaning that the related increase in JA would have a limited impact. Finally, with respect to the context in which a specific resource occurs, it should be recalled that our study was carried out during the COVID-19 pandemic. As social connection with others at work may be particularly relevant after a period in which social interactions were hampered by restrictions and social distancing [3], it is possible that workers were more willing to give up a degree of JA in favor of other resources, such as social support, in order to buttress good relations with their colleagues/supervisors and reduce professional and social isolation [154,155,156]. Of course, this does not imply that JA should no longer be regarded as a valuable resource. In the future, it could be useful to pay closer attention to new forms of JA aimed at integrating the modern organization of work, based on teamwork and interdependence (e.g., tied autonomy) [157], the specific needs of remote workers (e.g., a self-paced, self-determined use of new technologies) [158], and supervisors’ leadership styles (e.g., that value support and trust instead of excessive monitoring) [159,160]. 

We believe that our study has relevant practical implications for organizations and practitioners, in terms of primary and secondary prevention (e.g., directed to smart workers as a specific subpopulation of workers). First, by showing an association with SW and workload, this study suggests that hair cortisol/DHEA(S) ratio is a promising biomarker of WRS. The identification of a panel of possible biological indicators of WRS, similarly to the composite AL index [22,161], is a relevant goal for organizations and practitioners (e.g., occupational physicians) from the perspective of prevention and occupational health promotion. Notably, a strength of hair sampling is that it is a painless, noninvasive WRS assessment method that can also be performed by the workers themselves. Hence, the evaluation of hair cortisol/DHEA(S), coupled for example with the collection of other digital biomarkers (i.e., physiological data collected via digital devices, such as blood pressure and sleep quality) [162], could be useful to promote health (e.g., by fostering behavioral changes when working from home), as well as in terms of early detection and prevention of more severe consequences of WRS, among both in-person and remote workers [163]. Second, with respect to the potential detrimental effects of workload, organizations should encourage supervisors to learn new skills that will enable them to support smart workers in managing their job tasks when working remotely (e.g., e-leadership) [164], with a focus on mutual trust and instrumental/emotional support, rather than excessive monitoring and control [159]. Moreover, to reduce the tendency to exceed regular working hours and to forestall an “always-on culture” [165], specific organizational policies might be aimed at dissuading the use of work-related technologies (e.g., email, virtual meetings) during leisure time [166], including the acknowledgment of a right to disconnect [167]. Finally, in addition to these top-down strategies [168], interventions could also be aimed at promoting bottom-up, job-crafting interventions. For example, workers may be encouraged to proactively optimize their job [169] through increased structural job resources, such as seeking greater clarity of roles or tasks.

In addition to the aforementioned strong points, our study has some limitations. First, different forms of “remote working” are described in the literature, identified by terms such as telecommuting, remote work, telework, distance work and, clearly, SW. Although these phenomena overlap to some extent [4,5], some dissimilarities need to be acknowledged, with difficulties with respect to the evaluation of prior research and the generalization of results across different work arrangements and contexts [32]. Second, this study was carried out during the COVID-19 pandemic, during which the adoption of remote working was mostly imposed to contain the spread of SARS-CoV-2 and—in Italy—facilitated by ad hoc regulation. In this respect, we acknowledge that studies conducted prior to the COVID-19 pandemic have shown that SW can have positive outcomes, especially when adopted on a voluntary basis. For example, working from home voluntarily one or two days a week may allow employees to enjoy flexibility while maintaining face-to-face interactions with colleagues/supervisors, with positive consequences in terms of greater job satisfaction [70]. Contrarily, during the COVID-19 crisis, workers from private and public organizations were forced to suddenly work from home, often full-time. This condition of enforced SW during the pandemic may have exacerbated some negative features of SW, such as family–work conflict and increased social and professional isolation [12,170]. At the same time, although not voluntary, SW during the pandemic may also have had advantages, such as a lower risk of infection when working from home [171] or the possibility of keeping one’s job, avoiding furlough or layoff [172]. Again, this complex picture may pose a challenge to the interpretation of our findings. However, we believe our study to provide an insightful perspective on the work experiences and wellbeing of smart workers during the COVID-19 crisis, when millions of individuals worldwide were forced to work remotely in a “global experiment” of SW [3]. Third, in line with a considerable amount of past research, in this study we essentially compared in-person vs. remote workers with respect to their work experience and wellbeing. However, it should be recognized that SW is rarely an “all or nothing” phenomenon, as employees may differ with respect to the extent to which they work remotely [72]. Hence, future research should investigate whether and how the extent of remote working may affect individual and organizational outcomes such as work–life balance, wellbeing, and productivity. Fourth, this study included only two measurement occasions over a three-month time period. Although this is consistent with the rationale behind the research, future studies could be based on multiple, shorter intervals (i.e., shortitudinal design) [173]. This approach could be valuable to understand the unfolding of psychological and physiological responses over time, as well as to determine the optimal time interval for the process under investigation. Similarly, the repeated assessment of perceived job characteristics over time may have allowed the investigation of reciprocal associations. For example, in addition to the hypothesized relationships, it is also possible that employees experiencing psychophysical strain at T1 (i.e., those with a high hair cortisol/DHEA(S) ratio) could also report higher levels of job demands and lower levels of job resources at T2. Although these considerations are beyond the scope of this study, as they reflect different processes in the JD-R model, future research could explore these reciprocal relationships. Fifth, the study included a mixed group of workers, and the gender distribution was rather unbalanced (73.4% women), which may influence the generalizability of our results. While gender imbalance is neither uncommon in hair biomarker research [174] nor unexpected in the present study, given our research design (e.g., people with hair less than 3 cm long or who were bald could not be enrolled), further investigation on possible gender differences is warranted. Similarly, it should be acknowledged that SW may have different implications for different types of workers. For example, previous research has shown that managers experienced working from home to be more challenging than employees during the COVID-19 pandemic [175]. Therefore, given these potential limitations, further research is needed to replicate and extend the current findings. Finally, the role of personal demands (e.g., negative affectivity, perfectionism) and personal resources (e.g., self-efficacy and resilience) in the health impairment process of the JD-R model should be further examined [34,176,177,178,179,180].

## 5. Conclusions

Building on the JD-R and the AL models, this study showed that SW was negatively associated with hair cortisol/DHEA(S) ratio—a promising biomarker of WRS—over a three-month time period during the COVID-19 pandemic. At the same time, workload was positively associated with hair cortisol/DHEA(S) ratio over time among smart workers. Taken together, our findings suggest that SW can be conceived as a double-edged sword for workers’ wellbeing. On the one hand, some characteristics of SW associated with greater flexibility in defining work/life boundaries, likely related to a “bright side” of new technologies that facilitates techno-eustress [89], can help individuals to protect or replenish valuable resources, and to prevent negative health consequences over time [73,74]. On the other hand, other aspects of SW associated with information overload or disrupted workflow, possibly attributable to a “dark side” of new technologies linked to techno-distress [89], can lead to resource exhaustion and negative health consequences over time [73,74]. Furthermore, by integrating psychological and biological measures, our study contributed to shed light on the physiological processes potentially involved in the association between perceived aspects of the job (i.e., job demands/resources) and long-term health impairment—a theme on which the literature has urged additional research [34]. In terms of practical implications, this study suggests that the assessment of hair cortisol/DHEA(S) ratio, together with other—including digital—biomarkers, could prove to be a useful tool for occupational health protection and promotion. Additionally, concerning workplace interventions, our findings indicate that organizations and managers should shape a new way of working, tailored to remote employees’ specific needs and expectations (e.g., through revised organizational policies, support, and leadership styles). Finally, as SW will probably continue to play a central role after the COVID-19 pandemic, our study suggests an in-depth reflection on its broader meaning and effects on quality of life for people at work. While SW may contribute to “reinforce the self-image of responsible, committed, independent, and autonomous professionals/individuals” (p. 782) [29], it should be acknowledged that these beneficial resources would be available only to highly qualified employees who are able to work from home, thereby fostering inequalities with low-qualified workers, especially during crisis situations [181].

## Figures and Tables

**Table 1:** Multiple regression analyses for log cortisol/DHEA(S) ratio (Time 2): Model 1 and Model 2 (*n* = 124).

Predictors (Time 1)	Model 1		Model 2	
	*B*	*SE*	*B*	*SE*
Intercept	−0.360 ***	0.043	−0.362 ***	0.042
Gender ^1^	−0.133 ^†^	0.072	−0.140 ^†^	0.072
Age	0.008 **	0.002	0.007 **	0.002
Workload	0.032	0.029	−0.011	0.032
Job autonomy	0.009	0.021	0.008	0.023
Smart working ^2^	−0.177 **	0.067	−0.202 **	0.066
Workload x smart working			0.173 **	0.065
Job autonomy x smart working			0.031	0.048
Simple slope workload (in-person)			−0.011	0.032
Simple slope workload (smart working)			0.162 **	0.056
Total *R*^2^	0.186 ***		0.238 ***	
Change in *R*^2^			0.052 *	

Note: in all of the models tested, log cortisol/DHEA(S) ratio at Time 2 was the dependent variable. *B* = unstandardized regression coefficient; *SE* = standard error; *R*^2^ = squared multiple correlation. ^1^ Female = 0, male = 1; ^2^ in-person working = 0, smart working = 1. ^†^ *p* < 0.10. * *p* < 0.05. ** *p* < 0.01. *** *p* < 0.001.

## Data Availability

The data presented in this study are available upon reasonable request from the corresponding author. The data are not publicly available due to privacy reasons.

## Supplementary Materials

The following supporting information can be downloaded at: https://www.mdpi.com/article/10.3390/ijerph20136220/s1, Table S1: Multiple regression analyses for log cortisol/DHEA(S) ratio (Time 2): Model 1 and Model 2 (*n* = 124). References [115,117,122,123,124,126] are cited in the supplementary materials.
