# Supplementary material for: Is Smart Working Beneficial for Workers’ Wellbeing? A Longitudinal Investigation of Smart Working, Workload, and Hair Cortisol/Dehydroepiandrosterone Sulfate during the COVID-19 Pandemic
Source: Int J Environ Res Public Health. 2023 Jun 24;20(13):6220. doi: 10.3390/ijerph20136220 (PMC10341102; doi:10.3390/ijerph20136220)
Supplement: Supplementary file 1 [file ijerph-20-06220-s001.zip › ijerph-2303589-supplementary.pdf]

Supplementary Materials

Is Smart Working Beneficial for Workers' Wellbeing? A Longitudinal Investigation of Smart Working, Workload, and Hair Cortisol/Dehydroepiandrosterone Sulfate during the COVID-19 Pandemic

Alessandra Falco [1,*,†], Damiano Girardi [1,†], Achim Elfering [2], Tanja Peric [3], Isabella Pividori [3] and Laura Dal Corso [1]

1 FISPPA Section of Applied Psychology, University of Padua, 35131 Padua, Italy
2 Institute of Psychology, University of Bern, 3012 Bern, Switzerland
3 Department of Agricultural, Environmental and Animal Sciences, University of Udine, 33100 Udine, Italy
* Correspondence: alessandra.falco@unipd.it; Tel.: +0039-049-8276588
† These authors contributed equally to this work.

Method

Data Analysis

Since previous research has suggested an association between hair cortisol/dehydroepiandrosterone sulfate (DHEA(S)) concentrations and pregnancy status (D'Anna-Hernandez et al., 2011) as well as medication intake (Dettenborn et al., 2010), we further investigated the role of these variables. Additionally, the main models discussed in the manuscript, namely Model 1 (M1) and Model 2 (M2), were estimated controlling for the effects of pregnancy status and medication intake, where necessary. In M1, hair cortisol/DHEA(S) ratio at T2 was regressed on control and focal variables at T1. In M2, two interaction terms—between workload and smart working (SW), and between job autonomy (JA) and SW—were also included. As in the manuscript, the independent variables included in M1/M2 (excluding dichotomous variables) were mean-centered, to enable easier interpretations of results (Aiken & West, 1991). Additionally, a log-transformation of the cortisol/DHEA(S) ratio was used (Sollberger & Ehlert, 2016). Statistical analyses were conducted using R version 4.2.1 (R Core Team, 2022).

Results

Descriptive Statistics

Fifty-three participants (42.7%) were taking medication, while none were pregnant. Hence, pregnancy status was not considered further. Interestingly, there was no significant difference in log cortisol/DHEA(S) ratio between participants taking medication ($M = -0.41$, $SD = 0.36$) and those not taking medication ($M = -0.48$, $SD = 0.39$), $t(116.97) = -1.10$, $p = .27$, Cohen's $d = 0.20$.

Hypothesis Testing

Results of the regression analyses are presented in Table S1. After controlling for medication intake, the results did not change compared to the manuscript. In M1, the predictors at T1 accounted for 18.6% of the variance in the log cortisol/DHEA(S) ratio at T2 ($R^2 = 0.19$, $F(6, 117) = 4.46$, $p < .001$). In

this model, gender (*b* = −0.14, *SE* = 0.08, *p* = .08) and age (*b* = 0.01, *SE* = 0.00, *p* < .01) at T1 were associated with log cortisol/DHEA(S) ratio at T2, although the association was marginally significant in the former case. Medication intake at T1 was not associated with log cortisol/DHEA(S) ratio at T2 (*b* = −0.01, *SE* = 0.07, *p* = .92). Workload (*b* = 0.03, *SE* = 0.03, *p* = .27) and JA (*b* = 0.01, *SE* = 0.02, *p* = .68) at T1 were not associated with log cortisol/DHEA(S) ratio at T2. Smart working at T1 (0 = in-person working, 1 = smart working) was negatively associated with log cortisol/DHEA(S) ratio at T2 (*b* = −0.18, *SE* = 0.07, *p* = .01).

Table S1. Multiple regression analyses for log cortisol/DHEA(S) ratio (Time 2): Model 1 and Model 2 (*n* = 124).

Predictors (Time 1)	Model 1		Model 2	
	B	*SE*	*B*	*SE*
Intercept	−0.356***	0.056	−0.353***	0.054
Gender [1]	−0.136†	0.076	−0.145†	0.075
Age	0.008**	0.002	0.007**	0.002
Medication [2]	−0.007	0.068	−0.017	0.067
Workload	0.032	0.029	−0.011	0.033
Job autonomy	0.009	0.021	0.007	0.023
Smart working [3]	−0.177*	0.067	−0.203**	0.067
Workload x smart working			0.173**	0.065
Job autonomy x smart working			0.032	0.048
Simple slope workload (in-person)			−0.011	0.033
Simple slope workload (smart working)			0.163**	0.056
Total R^2	.186***		.238***	

Change in R^2	.052*

Note: log cortisol/DHEA(S) ratio Time 2 was the dependent variable in all the models tested. B = unstandardized regression coefficient; SE = standard error; R^2 = squared multiple correlation. [1] Female = 0, male = 1; [2] No medication = 0, medication = 1; [3] in-person working = 0, smart working = 1. †$p < .10$. *$p < .05$. **$p < .01$. ***$p < .001$.

In M2, the interaction terms accounted for an additional 5.2% of the variance in log cortisol/DHEA(S) ratio at T2, $F_{change}(2, 115) = 3.96$, $p = .02$, $f^2 = .07$ (Cohen, 1992). The interaction between workload and SW was significant ($b = 0.17$, $SE = 0.07$, $p < .01$), whereas the interaction between JA and SW was not ($b = 0.03$, $SE = 0.05$, $p = .51$). Simple slope analysis revealed that the association between workload at T1 and log cortisol/DHEA(S) ratio at T2 was positive and significant for smart workers ($b = 0.16$, $SE = 0.06$, $p < .01$), but not significant for in-person workers ($b = -0.01$, $SE = 0.03$, $p = .74$). Smart working strengthened the positive association between workload at T1 and log cortisol/DHEA(S) ratio at T2.

References

Aiken, L. S., & West, S. G. (1991). *Multiple regression: Testing and interpreting interactions*. Sage Publications, Inc.

Cohen, J. (1992). A power primer. *Psychological Bulletin*, *112*(1), 155–159. https://doi.org/10.1037/0033-2909.112.1.155

D’Anna-Hernandez, K. L., Ross, R. G., Natvig, C. L., & Laudenslager, M. L. (2011). Hair cortisol levels as a retrospective marker of hypothalamic–pituitary axis activity throughout pregnancy: Comparison to salivary cortisol. *Physiology & Behavior*, *104*(2), 348–353. https://doi.org/10.1016/j.physbeh.2011.02.041

Dettenborn, L., Tietze, A., Bruckner, F., & Kirschbaum, C. (2010). Higher cortisol content in hair among long-term unemployed individuals compared to controls. *Psychoneuroendocrinology*, *35*(9), 1404–1409. https://doi.org/10.1016/j.psyneuen.2010.04.006

R Core Team. (2022). *R: A Language and environment for statistical computing*. R Foundation for Statistical Computing. https://www.R-project.org/

Sollberger, S., & Ehlert, U. (2016). How to use and interpret hormone ratios. *Psychoneuroendocrinology*, *63*, 385–397. https://doi.org/10.1016/j.psyneuen.2015.09.031
